# Supplementary material for: Acceptability of short message service reminders as the support tool for PrEP adherence among young women in Mukono district, Uganda
Source: PLOS Glob Public Health. 2024 Jan 2;4(1):e0002492. doi: 10.1371/journal.pgph.0002492 (PMC10760663; doi:10.1371/journal.pgph.0002492)
Supplement: S1 Questionnaire — (PDF) [file pgph.0002492.s001.pdf]

Version number QE/02.0

Questionnaire

Study title: Acceptability of SMS reminders as an HIV PrEP adherence support tool and associated factors among Adolescent Girls and Young Women in Mukono district.

General information

1. **Participant ID NO**: **|___ |___| ___| ___|**
2. **Health facility providing PrEP: ______________________________**
3. **Interview Date**: **|___|___|/|___|___|/| ___| ___| ___| ___| [MM/DD/YYYY]**
4. **Name of the Interviewer: ____________________________**

Thanks for agreeing to participate. I will first begin by asking you some information about yourself.

A. Socio- demographic Characteristics

1. What is your Age in completed Years: |___|___|

In the following questions, check the appropriate box of the appropriate response (☒)

2. Where do you currently reside:

Village: |_____________________________________|

Parish: |_____________________________________|

Sub-county: |_____________________________________|

County: |_____________________________________|

District: |_____________________________________|

For how long have you resided at the mentioned address in (2) above?

|______|years and |________| Months

3. What is your current marital status?

1. |___|Married

2. |___|Single

3. |___|Cohabiting

4. |___|Divorced

5. |___|Separated

4. What is your religion?

1. |___|Anglican
2. |___|Catholic
3. |___|Moslem
4. |___|Born again
5. |___|Others. Specify |________________________|

5. What is your current Occupation?

1. |___|Formal Employed

2. |___|Self-Employed

3. |___|Housewife

4. |___|Unemployed

6. What is your completed highest level of education?

1. |___|No Education

2. |___|Primary level

3. |___|Secondary level

4. |___|Tertiary level

7. Are you currently in school?

1. |___| Yes

2. |___|No

B. Individual factors

8. When did you start using PrEP pills?

1. |___| Past 1 month

2. |___|Past 2 months

3. |___| Past 3 or more months

9. How many times have you visited the health facility for PrEP related issues in the past 3 month?

1. |___| Once

2. |___|Twice

3. |___| 3 or more times

10. Did you disclose use of Pre-Exposure Prophylaxis pills to anyone (like parent, spouse or peer)?

1. |___| Yes

2. |___|No

If No, go to question 13

11. To whom did you disclose PrEP use?

1. |___| Parent

2. |___|Spouse

3. |___| Peer/Friend

12. Did you get support in regard to taking your PrEP from the one you disclosed to (in question 9)?

1. |___| Yes

2. |___|No

13. Have you had any missed PrEP dose(s) in the past 4 weeks?

1. |___| Yes

2. |___|No

If no, go to question 15

14. What was the reason of missing the PrEP pills?

1. |___| Forgot

2. |___|Had no Pills

3. |___| Side effects

4. |___|Others. Specify |________________________________|

15. Have you moved away from your usual home for more than two consecutive days in the past 4 weeks?

1. |___| Yes

2. |___|No

16. Have you ever owned a personal mobile Phone?

1. |___| Yes

2. |___|No

17. Do you own a personal mobile phone currently?

1. |___| Yes

2. |___|No

18. Do you sometimes share your mobile phone with any other person?

1. |___| Yes

2. |___|No

19. Have you ever used a phone alarm in the past 4 weeks?

1. |___| Yes

2. |___|No

20. Have you ever read or sent an SMS on a mobile phone in the past 4 weeks?

1. |___| Yes

2. |___|No

21. Would you be willing to use SMS reminders as a PrEP adherence support tool?

1. |___| No

2. |___| Yes

if no, go to question 24

22. SMS reminders can be one-way where you only receive reminders or two-way where you can also reply and ask anything you want from a health care provider. Amongst the two methods of delivery of SMS reminders, which option would you prefer to use

1. |___| One way option

2. |___| Two way option

23. How often would you like to receive SMS reminders on your mobile phone?

1. |___| Daily

2. |___| Once a week

3. |___| Twice a week

4. |___| More than twice per week

5. |___| Once a month

6. |___|Others

24. Do you believe that use of SMS reminders as a PrEP adherence support tool leads to breach of your privacy?

1. |___| Yes

2. |___|No

C: Assessment of self-reported Literacy level on use of SMS reminders

**25. How often do you need someone to help you to read SMS?**	☐ 1. Never<br>☐ 2. Occasionally<br>☐ 3. Sometimes<br>☐ 4. Often<br>☐ 5. Always
**26. How often do you have challenges using SMS features on a mobile phone due to difficulty in understanding how they operate?**	☐ 1. Never<br>☐ 2. Occasionally<br>☐ 3. Sometimes<br>☐ 4. Often<br>☐ 5. Always
**27. How confident are you in Using SMS features by yourself?**	☐ 1. Extremely<br>☐ 2. Quite bit

	☐ 3. Somewhat<br><br>☐ 4. A little bit<br><br>☐ 5. Not at all
**Total score**	…………………….

D: Assessment of Self-reported Adherence to oral PrEP

**28. What has been your ability to take PrEP pills in the last 4 weeks?**	☐ 1. Very Poor<br><br>☐ 2. Poor<br><br>☐ 3. Fair<br><br>☐ 4. Good<br><br>☐ 5. Very Good<br><br>☐ 6. Excellent
**29. How often have you taken your PrEP pills all the time in the last 4 weeks?**	☐ 1. None of the time<br><br>☐ 2. A little of the time<br><br>☐ 3. Some of the time<br><br>☐ 4. A good bit of the time<br><br>☐ 5. Most of the time<br><br>☐ 6. All of the time
**30.*Now you will tell me the number of pills you have taken per week for the past 4 weeks***<br>**(a). How many pills have you taken in the past 7 days**	________________

**(b). How many pills did you take in the 7 days before last week above in (a) (7 days)**	________________
**(c). How many pills did you take in the 7days before the period mentioned above in (b) (14 days)**	________________
**(d). How many pills did you take in the 7days before the period above in (c) (21 days)**	________________
**Percentage of the days where the participant was able to take the PrEP pills exactly as directed in the last 4 weeks? (Scale of 0-100%)** *calculated from [(total pills taken/28)*100]*	________________
**Average percentage score**	………………………

E: SMS Reminder Acceptability Assessment tool (please check in the box provided with a cross (☒) for the participant responses)

**Question**	**Response**
**31. AFFECTIVE ATTITUDE**: How willing or unwilling are you to accept the use of SMS reminders as a PrEP adherence support tool?	☐ 1. Very unwilling<br>☐ 2. Unwilling<br>☐ 3. Neutral<br>☐ 4. Willing<br>☐ 5. Very willing
**32. BURDEN:** How much do you agree or disagree that the use of SMS reminders as a PrEP adherence support tool would be burdensome to you?	☐ 1. Strongly Agree<br>☐ 2. Agree

	☐ 3. Neutral<br>☐ 4. Disagree<br>☐ 5. Strongly Disagree
**33. ETHICALITY:** To what extent do you think use of SMS reminders as a PrEP adherence support tool aligns with your values and beliefs?	☐ 1. Very inappropriate<br>☐ 2. Inappropriate<br>☐ 3. Neutral<br>☐ 4. Appropriate<br>☐ 5. Very appropriate
**34. INTERVENTION COHERENCE:** To what extent do you agree or disagree that you understand how SMS reminders can be used to support oral PrEP adherence?	☐ 1. Strongly Disagree<br>☐ 2. Disagree<br>☐ 3. Neutral<br>☐ 4. Agree<br>☐ 5. Strongly Agree
**The following questions inquire about what you think of use of SMS reminders as a PrEP adherence support tool**	
**35**. **OPPORTUNITY COSTS:** To what extent do you agree or disagree that you would give up some of your time to use SMS reminders as a PrEP adherence support tool?	☐ 1. Strongly Disagree<br>☐ 2. Disagree<br>☐ 3. Neutral<br>☐ 4. Agree<br>☐ 5. Strongly Agree
**36**. **PERCEIVED EFFECTIVENESS:** To what extent do you agree or disagree that SMS reminders would help you in adhering	☐ 1. Strongly Disagree<br>☐ 2. Disagree

to oral PrEP?	☐ 3. Neutral<br>☐ 4. Agree<br>☐ 5. Strongly Agree
**37. SELF-EFFICACY:** To what extent do you agree or disagree that you would confidently utilize SMS reminders as a PrEP adherence support tool?	☐ 1. Strongly Disagree<br>☐ 2. Disagree<br>☐ 3. Neutral<br>☐ 4. Agree<br>☐ 5. Strongly Agree
**Total score**	…………………………

Thank you for participating in this interview
