# Supplementary material for: Acceptability of short message service reminders as the support tool for PrEP adherence among young women in Mukono district, Uganda
Source: PLOS Glob Public Health. 2024 Jan 2;4(1):e0002492. doi: 10.1371/journal.pgph.0002492 (PMC10760663; doi:10.1371/journal.pgph.0002492)
Supplement: Attachment — Submitted filename: Response_to_Reviwers_2.pdf [file pgph.0002492.s003.pdf]

MUTEEBWA LABAN

P. O. BOX 500269, Kampala Uganda

Email: mutlabans@gmail.com

11th November 2023.

To: The Editor-In-Chief

PLOSE Global Public Health

Dear Sir/ Madam

RE: Response to Comments in regard to the Manuscript titled "Acceptability of short message service reminders as the support tool for PrEP adherence among young women in Mukono district, Uganda".

Thank you so much for the additional comment.

We have addressed these comments point by point as described below. The cited line numbers correspond to the Revised Manuscript with tracked changes

There is need to do more to acknowledge your study limitations

The limitations section has been updated. A possible source of misclassification bias resulting from concurrent measurement of exposure and outcome was acknowledged (see line 250-251)

Please add more detail to the inclusion criteria for your study

The eligibility criteria have been revised. See line 94-96

One of the reviewers has requested that you attach the study questionnaire.

The questionnaire has been added as "supporting information".

We look forward to your feedback, and we are happy to respond to any other comments that come up

Thank you again for considering this manuscript under your review.

Sincerely

Muteebwa Laban
