# Supplementary material for: Acceptability of short message service reminders as the support tool for PrEP adherence among young women in Mukono district, Uganda
Source: PLOS Glob Public Health. 2024 Jan 2;4(1):e0002492. doi: 10.1371/journal.pgph.0002492 (PMC10760663; doi:10.1371/journal.pgph.0002492)
Supplement: Attachment — Submitted filename: Response to Reviewers.docx [file pgph.0002492.s002.docx]

MUTEEBWA LABAN

P. O. BOX 500269, Kampala Uganda

Email: mutlabans@gmail.com

18^th^ October 2023.

To: The Editor-In-Chief

PLOSE Global Public Health

Dear Sir/ Madam

**RE: Response to Comments in regard to the Manuscript titled “Acceptability of short message service reminders as the support tool for PrEP adherence among young women in Mukono district, Uganda”.**

Following the review of the above mentioned manuscript, the reviewers raised some comments to address.

We have addressed these comments point by point as described below. The cited line numbers correspond to the Revised Manuscript with tracked changes

**Reviewer #1: Although technology-based interventions have been recommended to support the effective use of daily oral PrEP among AGYW, few studies have assessed the acceptability of technology-based interventions such as SMS among AGYW. The study includes relevant information to fill this existing gap. The authors can consider the following items:**

**Introduction
• Line 47- suggest removing “taking PrEP” as already stated in the sentence.**

The words “taking PrEP” have been deleted. See line 47

**• Lines 60-61: the data provided is from 2014. Any more up-to-date data?**

Section updated with recent (2021) population projections from the Uganda Bureau of Statistics. See line 76

**Methods
• Was this cross-sectional study conducted in DREAMS-supported sites? If so, suggest stating this information to provide additional context.**

The statement “*MUWRP implements PEPFAR’s “Determined, Resilient, Empowered, AIDS-free, Mentored and Safe” (DREAMS) project across the district with an overarching goal of reducing the incidence of HIV among AGYW through provision of health services, enhancing social skills, economic empowerment and business start-ups, AGYW in this program access HIV prevention services including PrEP*” has been added from line 73-78 to provide more context on the DREAMS program”. See line 81-85

**• Inclusion criteria- specify if you are referring to at least one month of daily oral PrEP (versus stating PrEP alone).**

We have rephrased the criterion to *“at least one month since the start of oral PrEP”* (line 89-91).

We have added a statement *“We recruited 142 AGYW who are current participants in the PEPFAR DREAMS project”* to clarify that these participants were also DREAMS participants. Line 82

**• Suggest including more information on how AGYW were recruited to complete the survey. Was it a convenient sample of AGYW from across a specific number of DREAMS supported sites or safe places? Please provide further information on how AGYW were recruited.**

We have rephrased the statement to give more information as quoted below; *“The AGYW in the DREAMS project met at the designated safe spaces once a week for project-related activities. The study team visited all safe spaces on the meeting day of each of the groups and AGYW with PrEP prescriptions were invited to participate in the study through their peer-leaders. The questionnaire was administered by trained research assistants to consecutively enrolled AGYW until the required sample size was met. The study was conducted between 1^st^ and 30^th^ August 2023”.* See line 97-102

**• Please provide more information about the acceptability of SMS reminders. Did the researchers use a validated scale? If so, specify and state if previously used to assess the acceptability of technology-based interventions for PrEP.**

The Theoretical Framework of Acceptability (TFA) has been used in similar populations elsewhere in Sub-Saharan Africa (reference provided) and has been consistently t was highly reliable in measuring acceptability in this population with a reliability coefficient 0.895.See line 110. This was declared in the limitation section.

**• Line 96: “Participants with a score above 21 were categorized as having accepted SMS reminders”. Please provide information for selecting this specific threshold.**

We have included a reference to support the cut-off. Briefly, literature shows that where no known cutoffs exist to categorize outcomes, then percentiles can offer an approach to set the thresholds of continuous outcomes for which categories can be deduced. The citation for previous work has been added. See line 117-118

**• Line 103- “We analyzed data in STATA…”. Suggest including this sentence as the last sentence of the data analysis.**

We have included a statement highlighting the software we used. “We analyzed data in STATA version 17.0 (Texas, USA)”. See line 138

**• Lines 109- “We used the modified Poisson regression with robust standard of errors”. The measure of effect is prevalence ratio. Any reasons for not using multivariable log-binomial regression model?**

We opted for the modified Poisson regression model following non-convergence of the log binomial regression model. Also, estimates of the prevalence ratio and the corresponding 95% confidence intervals given by the log binomial were similar to the estimates from the modified Poisson regression model with robust standard errors so was a good alternative for performing our analysis.

**Results
• Suggest removing the term “slightly” when presenting the results.**

We have deleted the word “slightly”. See lines 152

**• Lines 137 “Of those who owned a mobile phone, almost two thirds reported that they share it with another person (64.9% (74/114).” This information contradicts the values in the table. Revise one of them for consistency.**

We have corrected this to be *“Of those who owned a mobile phone, almost a third reported that they shared it with another person (35.1% (40/114))”.* See lines 159 to 160

**• Suggest removing the number of individuals in the title of your table. This information is included in the tables.**

We have deleted the number of individuals removed from the tittle of all tables.

**• Tables- for consistency, use the same number of decimals (e.g.2) for all numbers**

In the tables, percentages are consistently with one decimal place, while the prevalence ratios and their respective confidence intervals are consistently with two decimal places. The P values are consistently with three decimal places (since level of significance is at 0.05).
 **Discussion
• General comments on the discussion: Suggest revising the discussion to compare/contrast your results to other studies related to PrEP among AGYW versus expanding HIV treatment among different populations (as much as possible). Currently, the discussion includes few references, and many of them are not directly related to PrEP, technology-based interventions and AGYW.**

Just say we have enriched the discussion section to include more recent studies conducted among similar populations.

**• Paragraph 206-208- suggest rephrasing or removing this paragraph as it does not add to the study and also claims are not well supported by the scientific literature.**

Paragraph 206-208 has a statement *“In this study, AGYW who stayed in rural settings were 0.92 times less likely to accept the SMS reminders as a PrEP adherence support tool compared to those who stay in urban settings”* which is an interpretation of the findings in our study (refer to results table 3).

**• Suggest reviewing the limitations. Some additional limitations to consider (1) the study focuses on the perceived acceptability of SMS which may differ from the acceptability in real-life once AGYW receive SMS for PrEP support; (2) the survey seems to have recruited a convenient sample of AGYW (not randomly selected); and (3) the survey did not seems to have used validated scale among the study population.**

The statements *“A non-probability sampling approach (consecutive sampling) was used to select study participants, thus this may not have been a representative sample much more than half (58.2%) of the registered PrEP users were involved in the study. The TFA was not previously validated among AGYW to measure acceptability, however it was highly reliable (reliability coefficient was 0.895). Nonetheless, our study reported perceived acceptability of SMS reminders which could be different form the actual acceptability following deployment of the intervention. However, our study provides key information that can guide the packaging of the SMS reminder intervention and subsequent implementation*”, were added to the limitations section to reflect the additional limitations of the study.

**Reviewer #2: Overall, the paper gives great insights into SMS acceptability as a support tool for PrEP use among young women. The author may consider the following comments;**

**Line 30-31: Please specify the direction of the association of these two variables.**

Statement *“Rural residence (aPR: 0.92, 95% CI (0.84, 0.99)) and having belief that SMS cannot breach individual’s privacy (aPR: 1.40, 95% CI (1.07, 1.84)) were significantly associated with acceptability of SMS reminders”* has been changed to *“Rural residence was negatively associated with acceptability of SMS reminders (aPR: 0.92, 95% CI (0.84, 0.99)) and having belief that SMS cannot breach individual’s privacy (aPR: 1.40, 95% CI (1.07, 1.84)) was positively associated with acceptability of SMS reminders”*. See line 31-33

This reflects the direction of the prevalence ratios

**Line 57: It may be important for the reader to know why AGYW has increased levels of stigma compared to other populations.**

Statement *“because they are in a developmental transition and in a complex family web”* has been added. See line 63-64

**The author states in the abstract that TFA was used, may be important to mention briefly in the background what it is and why it was important and relevant to use it when introducing your study in the final paragraph.**

Statement *“Moreover, most studies have measured acceptability of SMS reminders, using a single question measure which may carry a lot of bias. Measuring this complex attribute using a composite question approach like utilizing the Theoretical Framework of Acceptability (TFA) may provide valid estimates of acceptability. The TFA has seven constructs that all measures important components that influence the overall acceptability of such a technology-based intervention”* has been added in the background section to introduce the framework. See line 54-59

Further information on how the framework was used in this study is highlighted in the methods section (subsection: study variables). See line 117-118

**Line 69: The author should consider providing citations for these statements: The district has a population of 599,817 people of whom about 187,143 are aged 10 to 24 years. The HIV prevalence among persons aged 15 years and above in Mukono district is 6.1% which is higher than the national prevalence (5.4%) thus is a priority district for HIV prevention.**

We have included citations for the statistics. See line 76

**Line 81: Saying the study had no exclusion criteria may mean anyone could possibly participate in the study yet you outline the inclusion criteria. The author may consider stating that individuals who did not meet these criteria were excluded.**

The exclusion criteria are not the opposite of the inclusion criteria, rather they consist of the characteristics of the individuals that already fit in the inclusion criteria that might be excluded from participation. From this background no such characteristics were used to exclude the participants that fit the inclusion criteria

**Line 93: Consider attaching the questionnaire as a supplement tool or mention the kind of question the participants were asked for the reader to understand.
In the results sections, ideally, the second table should show the main findings of your study. Consider providing a table or figure that shows acceptability. Although the reported individual characteristics are important, it is essential to tell the reader your key findings early in your result section.**

The main finding on level of acceptability was just a single proportion stated in-test with its respective confidence interval and thus didn’t warrant use of a table. See line

**Line 164-166: On factors associated with acceptability, the author just mentions that there is an association, it is important to give the direction of the association is it positive or negative for clarity. Is it increasing the likelihood of acceptability of SMS or is it reducing the likelihood? Also, consider stating that there were no other variables associated with the SMS acceptability.**

The direction of association was updated. See line 186-188

**Line 169: The asterick in Table 3 doesn’t appear anywhere in the table. Also, is there an explanation why (n) in the acceptability result is different with the (n) in your demographics?**

The astericks have been clearly labeled. See table 3

The (n) is the proportion of study participants who accepted SMS reminders by the respective levels of independent variables.

**In the discussion, the author has done well in reporting other studies and reviewing literature but has failed to give strength to their own findings. First, the first paragraph in your discussion does not fully summarize your findings. Second, it’s important to be clear on what you found and how it is relevant to other studies. There are different significant aspects of your findings that are not clear in your discussion. For instance, there was a significant association between rural areas and breach of privacy how does that compare with other literature? There were also preferences in either one or two-way SMS how does that compare?**

First paragraph has been updated to include all findings. Statement “*Moreover, individuals from rural settings were less likely to accept SMS reminders, while those that believe that SMS reminders cannot breach their privacy were more likely to accept this intervention”* added on the first paragraph to complete summary of the findings.

**Paragraph 3 & 4 of the discussion section discusses the significant factors (residence and breach of privacy)**

We have updated the discussion section.

**Line 188: In this study, the behavioral changes they targeted didn’t involve a lot of stigma like that associated with the use of PrEP. What does this mean and what behavioral changes are implied here?**

This statement was deleted

**Line 201 Motivation for taking ART in HIV-negative AGYW may be confusing to the reader the author may consider just calling it PrEP.**

We have deleted the statement *“where the goal is prevention of HIV*” and replaced with *“as PrEP to prevent HIV*”.

**Line 203: Also, in the China study the youth were underrepresented yet they may have varying levels of acceptability of SMS reminders compared to the adults. This statement is unclear, how were they underrepresented? and in which context? Also, provide a citation.**

The study referred to recruited both young adults (18-24 years) and older people (≥25 years), but not adolescents. We have provided the citation for the study. .

**Line 212: It would only be fair to compare with the same population I think adults’ level of acceptability may differ from AGYW.**

As highlighted above (and a guiding statement at the start of discussion section sentence 4), there is limited literature regarding acceptability of SMS reminders among AGYW taking PrEP, comparing with a population taking antiretroviral therapy for HIV treatment may provide a proxy to what factors may influence acceptability of the intervention in this population

**Line 206: Specify the value in % for clarity.**

Not clear of the percentage referred to. Only the interpretation on the prevalence ratio is presented. The proportion of the individuals who stayed in rural setting is presented in results section for reference

**Paragraph 206: The author states that in Uganda most population are from rural area, What is the implication of this given that the rural area is less likely to accept SMS? Consider reviewing studies showing that SMS helps in adherence and how that may have a potential impact on rural residents.**

Statement “*This could have far reaching impact on the acceptability of SMS reminder intervention. Therefore AGYW living in such rural settings may require additional support to use this intervention”*, added to emphasize the implication of the finding of the study. See line 237-239

**Line 213: Clarify how social economic differences impact either acceptance or none acceptance of the SMS**

The socio-economic status of China (both rural and urban) which is a high income country is far much different Uganda which is a low income country

**Paragraph 217: Consider reviewing studies that have assessed the outcome of breach of confidentiality in PrEP use especially among young women and not just PLHIV.**

Paragraph updated

**Line 224: It needs to come out clearly in your method section that some data were collected retrospectively.**

Variables that collected information retrospectively have been indicated in the section for variables (in methods). See line 121

We thank you so much for the very insightful and valuable comments that were provided aimed to improve the quality of the paper.

We look forward to your feedback, and we are happy to respond to any other comments that come up

Thank you again for considering this manuscript under your review.

Sincerely

Muteebwa Laban
